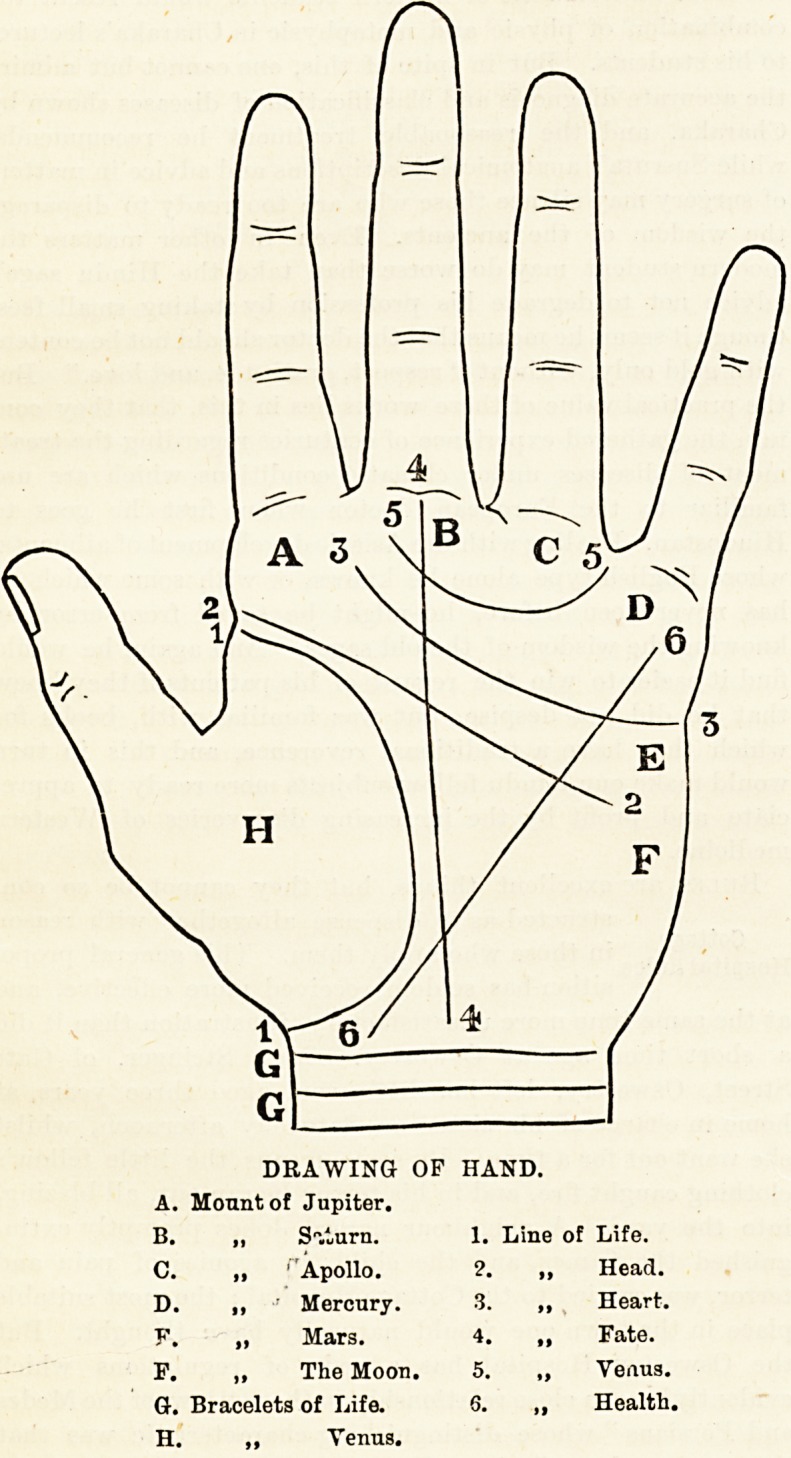# Palmistry Notes

**Published:** 1888-02-04

**Authors:** 


### Amusements for Convalescents.

### PALMISTRY NOTES.

By a Lady.

(*Continued from page* 230.)

DRAWING OF HAND.

| | | | |
|---|---|---|---|
| A. Mount of Jupiter. | | | |
| B. „ Saturn. | | 1. Line of | Life. |
| C. „ Apollo. | | 2. „ | Head. |
| D. „ Mercury. | | 3. „ | Heart. |
| F. „ Mars. | | 4. „ | Fate. |
| F. „ The Moon. | | 5. „ | Venus. |
| G. Bracelets of Life. | | 6. „ | Health. |
| H. „ Venus. | | | |

### THE LINES OF THE PALM.

In the palms of our hands we shall find marked with more or less distinctness four lines, to which the names of Life (1), Head (2), Heart (3), Fate (4), are given. The two other lines —Health (6), and Venus (5)—are not found in every hand. In the drawing before us all the lines are clearly delineated. Let us begin with the most important line in the hand, *the Life Line* (1), encircling the ball of the thumb, and continuing its course to the wrist. From the length and clearness of this line we judge of the possible duration of our existence, of how it will fare with us in sickness and in health, and of whether our life will be one of ease or of anxiety. It goes almost without saying that a long, firmly-marked line indicates a long life, blessed with good health. A good disposition is shown also by this formation : if, instead of being clear and even, the line is thick and irregular, it denotes an envious and capricious disposition and uncertain health. Sometimes a second line is found *inside* the Life Line, following its whole course ; this is indicative of wealth and distinction, and it will protect us in the dangers foreshadowed by breaks or crosses on the main line.

*The Line of Life* (1) *should unite with the Head Line* (2) *at its outset.* This gives us foresight and prudence in the conduct of our worldly affairs. Parted from the head line, we shall be self-willed and impulsive, with a tendency to vanity and exaggeration, which will lead to untruthfulness if the space between the lines is filled with many little cross bars. Sometimes instead of starting from the edge of the hand, the line commences under Jupiter *(a)*; this prognosticates renown and gratified ambition. The union of the Lines of Life (1), Head (2), and Heart (3), beneath the first finger, fortunately of very rare occurrence, points to some grave disaster, possibly death from an accident or by the hand of an assassin. All upward branches from a line are good; from the Line of Life they indicate wealth and ambition; if continued through other lines the good prognosticated will result from our own exertions. Downward branches are not lucky; they denote uncertain health, and vicissitudes occasioned by pecuniary reverses. A fork at the beginning of the line signifies vanity; but further on, under Jupiter, it denotes fidelity. When found on the centre of the line, a fork shows diminished mental force, which must be regarded, and care must be taken not to overtax the brain at the period where this bifurcation is apparent. By making a mental calculation, placing an imaginary figure of 50 on the middle of the line, and dividing the first half into small periods of ten years, till we reach this figure, and the remaining half in the same manner from the centre to the end of the line, which, if it curve right round the thumb, will bring us to the total of 100 years. Few Life lines extend to this period, and by deducting 10, 20, or more years till we arrive at the point where the Line ceases, we are able to arrive at the possible duration of our life.

The sudden termination of the line is an indication of death; a break indicates an illness; and if the line is broken in *both hands* there is fear of a fatal termination; but to make this prognostication *absolute*, the line must be broken in *both hands* at the same point. This applies to all lines and marks wherever found. Significant lines in one hand only betray a *possibility* of the event befalling; to become a *certainty* they must be found in both hands. A small line just cutting the Line of Life premises marriage at the age when it appears; and a line from the Mount of Venus (*h*) continued to Apollo (*c*) gives us artistic celebrity. If the Life Line is frayed at the extremity, it points to reverses and hard work in old age. Coming out boldly into the palm is a sign of long life; lying too near the thumb signifies weakness. Upward lines on to Jupiter (*a*) indicate pride and ambition, and when a star appears on the Mount it shows that our ambition will be gratified.

*Line of the Head* (2).—This line should join the Life Line (1) under the first finger, and from thence trace a clear line across the hand to the Mount of Mars (*e*). This formation gives us intelligence, with prudence, caution, and self-control in the exercise of it, calmness in danger and resource in moments of emergency. A really good, firm line of the head will go far to mitigate the sinister effects of any bad or weak sign in a hand by giving energy and resisting powers to the character. To a much-rayed palm, a good head line, forked at the extremity, with a strong line of Apollo (*c*), gives great ability to the owner. If the line is united to the Life Line for some distance, it indicates want of confidence and excessive shyness.

*The Head Line parted from the Life Line*, and taking, as it were, an independent course from its outset, indicates impetuosity, self-assertion, with a tendency to jump to conclusions, which would land the owner in frequent difficulties, were it not that the boldness and audacity inseparable from these qualities enable him to extricate himself with ease from the many little embarrassments into which this rashness and want of forethought would otherwise lead him. In the hand of an actor or public man this audacity will serve its owner in good stead, making him ever ready with a speech or action befitting the occasion, more particularly if the line terminates in a fork.

(*To be continued.*)

## Figures and Tables

**Figure f1:**